# Supplementary material for: Targeting the cochlin/SFRP1/CaMKII axis in the ocular posterior pole prevents the progression of nonpathologic myopia
Source: Commun Biol. 2023 Aug 29;6:884. doi: 10.1038/s42003-023-05267-2 (PMC10465513; doi:10.1038/s42003-023-05267-2)
Supplement: Supplementary file 2 — Supplementary Figures [file 42003_2023_5267_MOESM2_ESM.pdf]

Supplementary figures

S.1

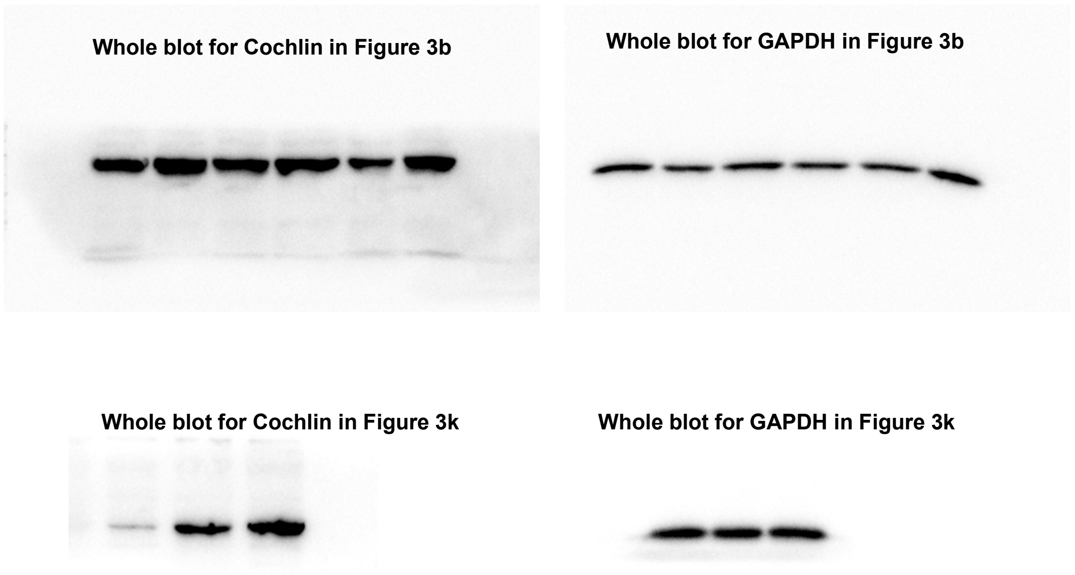

Supplementary Fig. 1 Whole blots for Figure 3b and 3k.

S.2

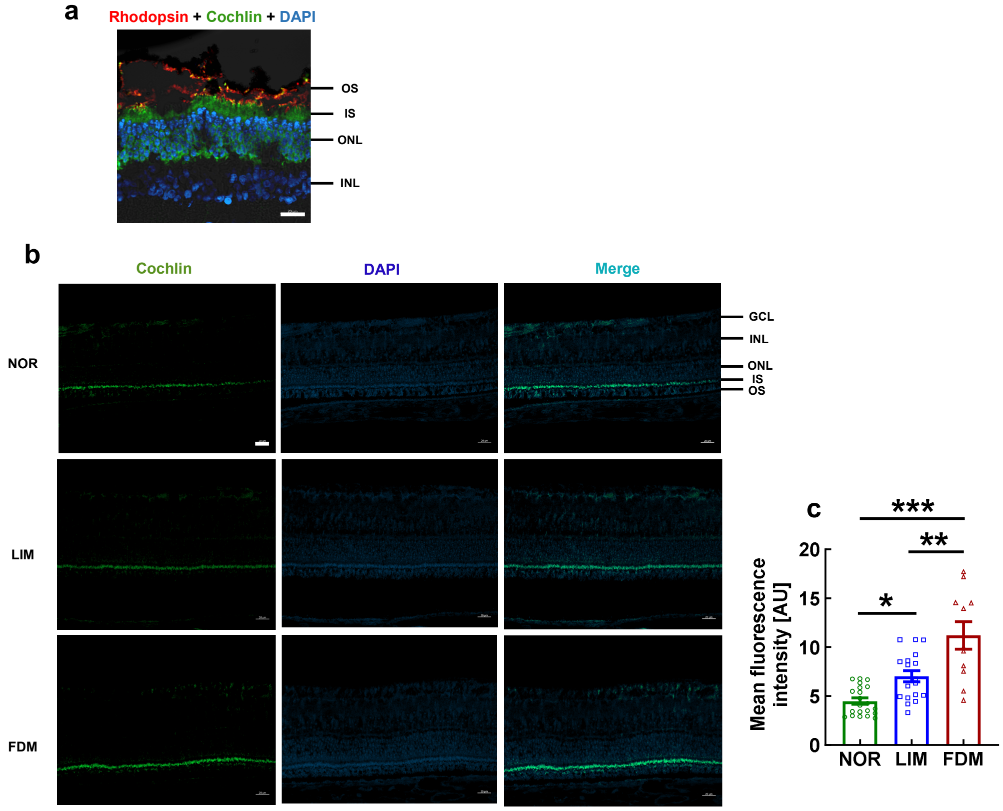

Supplementary Fig. 2 Immunofluorescence staining of cochlin in the retinas of guinea pig myopia models. A representative picture of double immunostaining is shown in (a). The green fluorescence signal (cochlin) was primarily localized at the outer nuclear layer and the inner segments of retinal photoreceptors, with sparse distribution in the outer segments. The red fluorescence signal (rhodopsin) was localized at the outer segments of retinal photoreceptors, with dispersed colocalization with the green signal (cochlin). Representative pictures of cochlin immunofluorescence staining in normal and myopic retinas are shown in (b). The intensity of the green fluorescence was quantified in (c) and compared using one-way ANOVA (n=11-21). All data represent the mean±SEM. * $P < 0.05$, ** $P < 0.01$, *** $P < 0.001$. GCL: ganglion cell layer; INL: inner nuclear layer; ONL: outer nuclear layer; IS: inner segment; OS: outer segment.

S.3

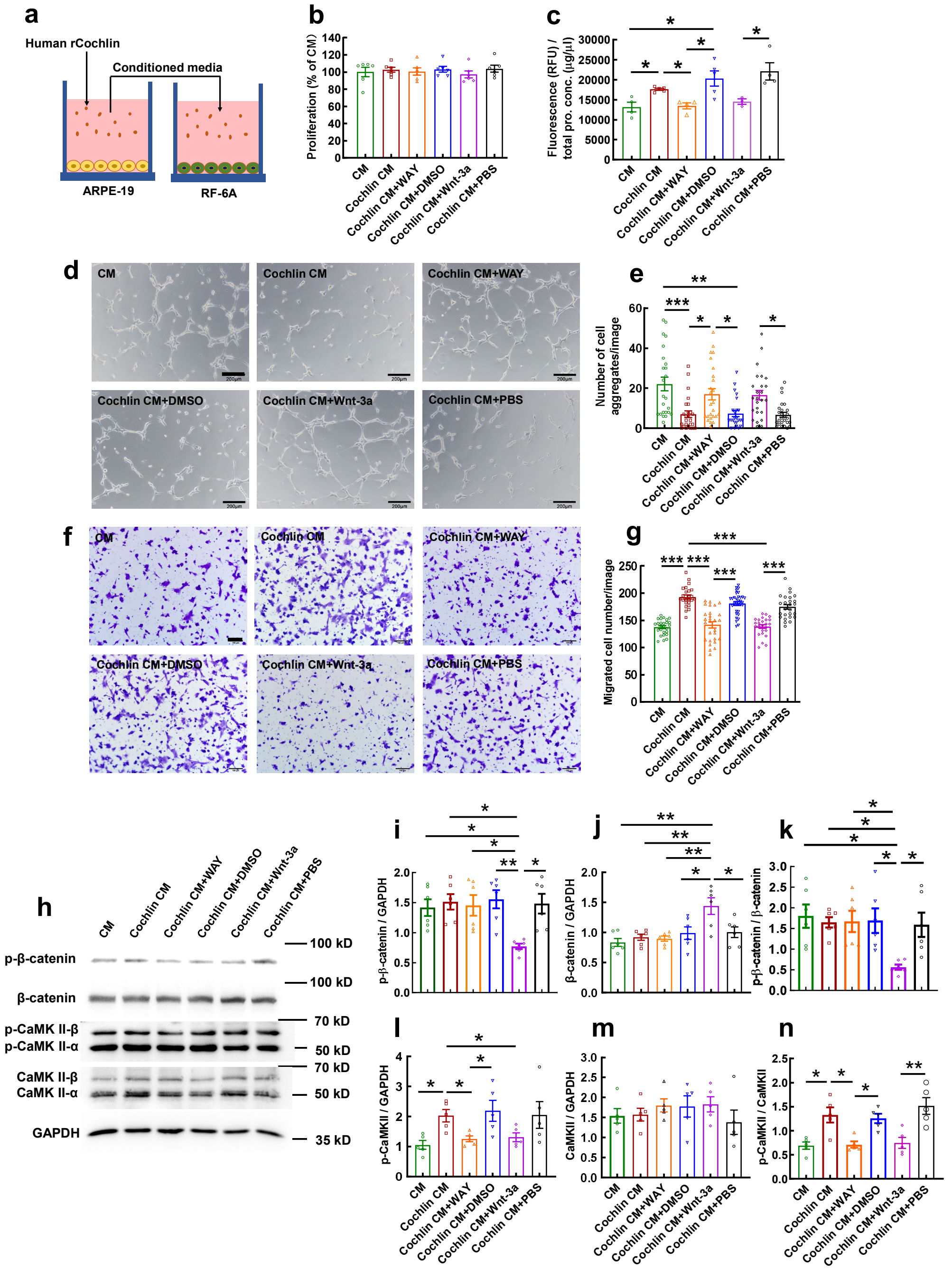

Supplementary Fig. 3 Conditioned media from cochlin-treated ARPE-19 cells caused dysfunction of choroidal vascular endothelial cells through noncanonical Wnt/CaMKII signaling. The experimental rationale is illustrated in (a). The effects of CM on the proliferation of choroidal vascular endothelial cells were examined by a CCK-8 assay (b) and compared using one-way ANOVA (n=6). The

effects of CM on the apoptosis of choroidal vascular endothelial cells were examined by a caspase 3/7 activity assay (c) and compared using one-way ANOVA (n=3-5). Representative images of the Matrigel assay of CM-treated choroidal vascular endothelial cells (d). Scale bar = 200 μm. Quantification of the Matrigel assay (e) was compared with one-way ANOVA (n=24). Representative images of the Transwell assay of CM-treated choroidal vascular endothelial cells (f). Scale bar = 100 μm. Quantification of the Transwell assay (g) was compared with one-way ANOVA (n=24-32). Representative western blots of p-β-catenin, β-catenin, p-CaMKII, and CaMKII in CM-treated choroidal vascular endothelial cells (h). Quantification of the western blots (i-n) was compared using one-way ANOVA (n=5 for p-β-catenin and β-catenin; n=6 for p-CaMKII and CaMKII). All data represent the mean ± SEM. * $P < 0.05$, ** $P < 0.01$, *** $P < 0.001$. RPE: retinal pigment epithelium; CM: conditioned media. CCK: Cell Counting Kit. CaMKII: Ca^{2+}/calmodulin-dependent protein kinase II.

S.4

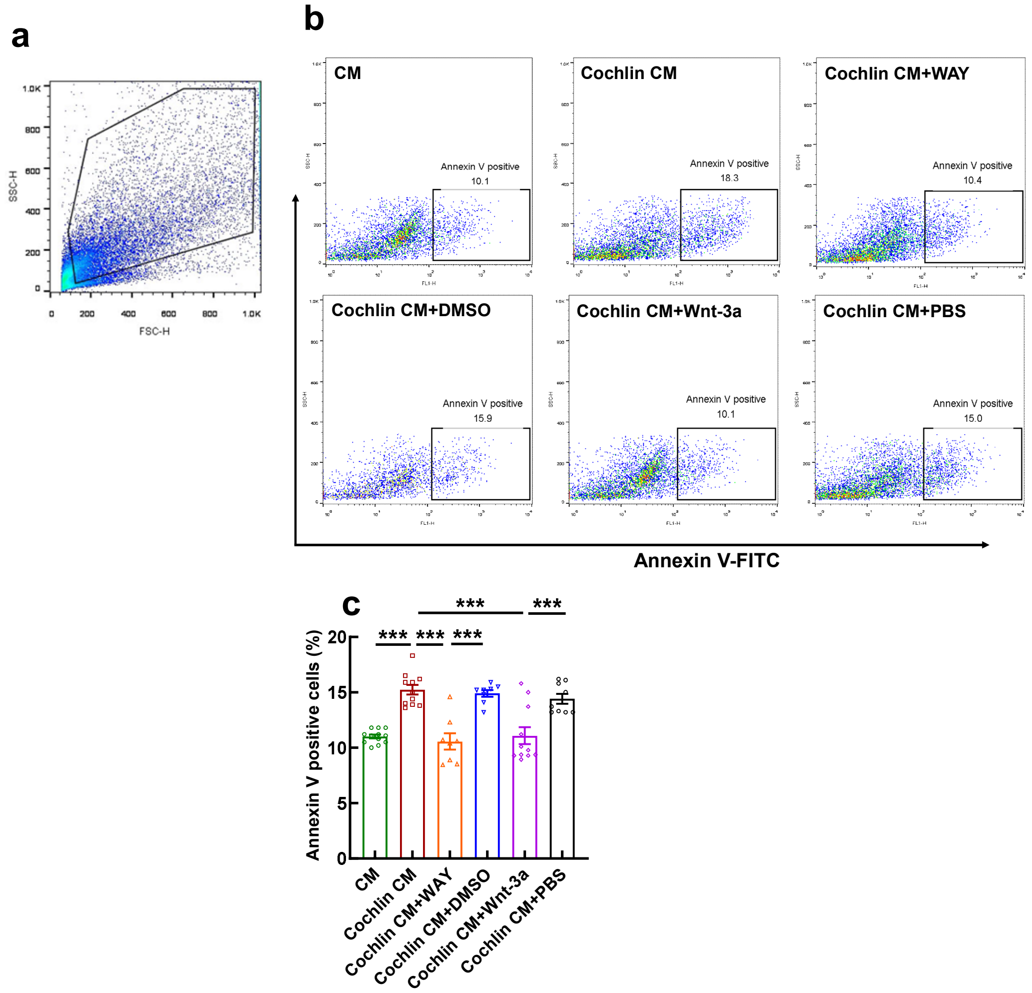

Supplementary Fig. 4 The effects of conditioned media on the apoptosis of choroidal vascular endothelial cells were examined by Annexin V staining and flow cytometry. The gating strategy of unstained RF/6A cells is shown in (a). Representative flow cytometry images of different cell groups are shown in (b). The flow cytometry analysis is quantified in (c) and compared among the experimental cell groups using one-way ANOVA (n=8-12). *** $P < 0.001$. CM: conditioned media; DMSO: dimethyl sulfoxide; PBS: phosphate buffered saline.

S.5

Whole blot for p-β-catenin in Supplementary figure 3h

Whole blot for β-catenin in Supplementary figure 3h

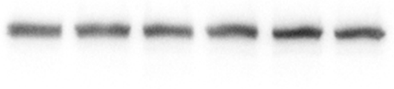

Whole blot for p-CaMKII in Supplementary figure 3h

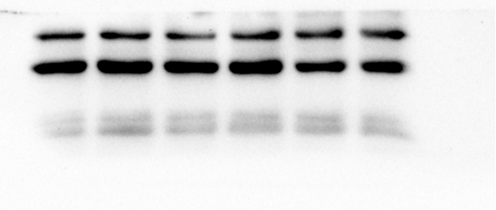

Whole blot for CaMKII in Supplementary figure 3h

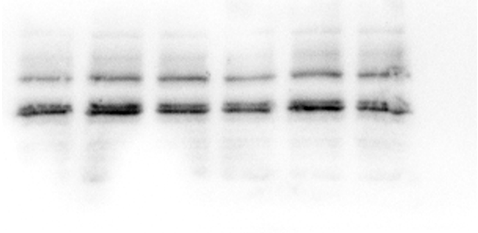

Whole blot for GAPDH in Supplementary figure 3h

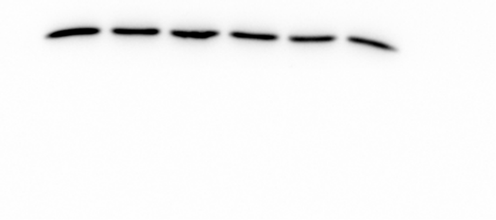

Supplementary Fig. 5 Whole blots for Supplementary Fig. 3h.

S.6

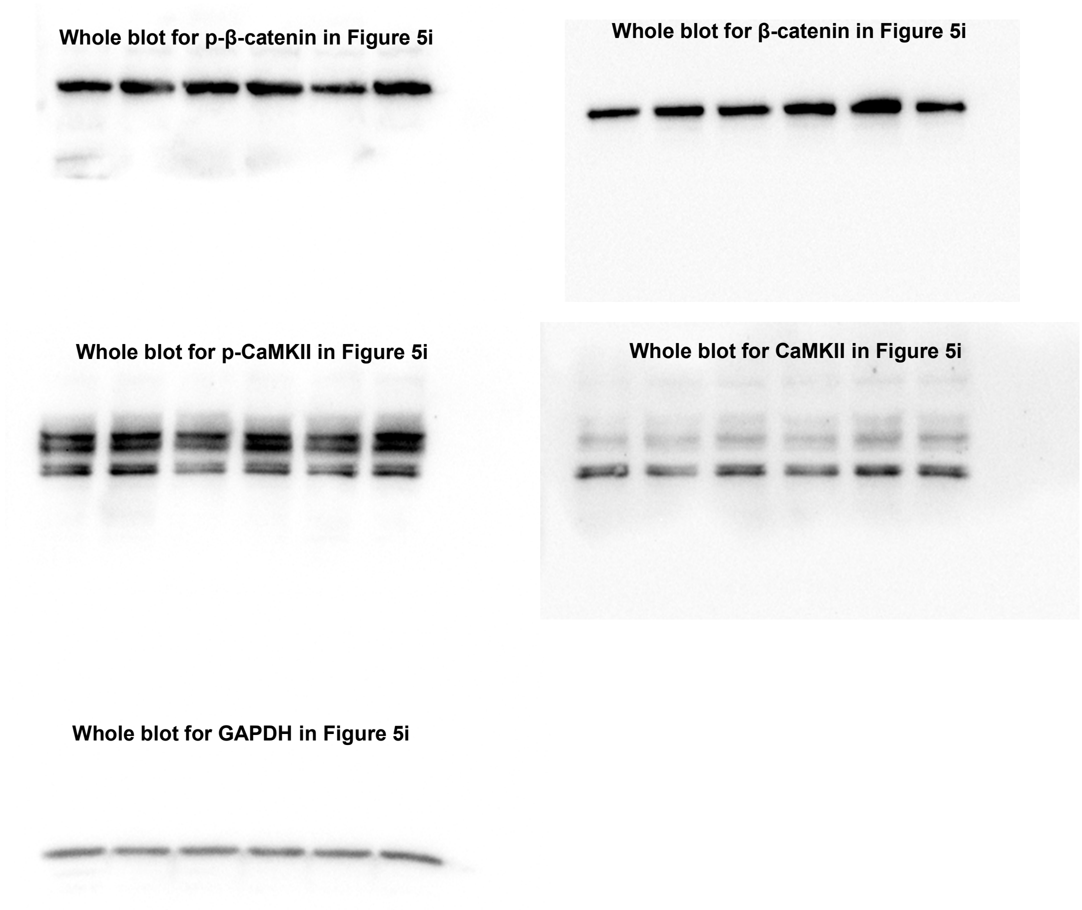

Supplementary Fig. 6 Whole blots for Fig. 5i.

S.7

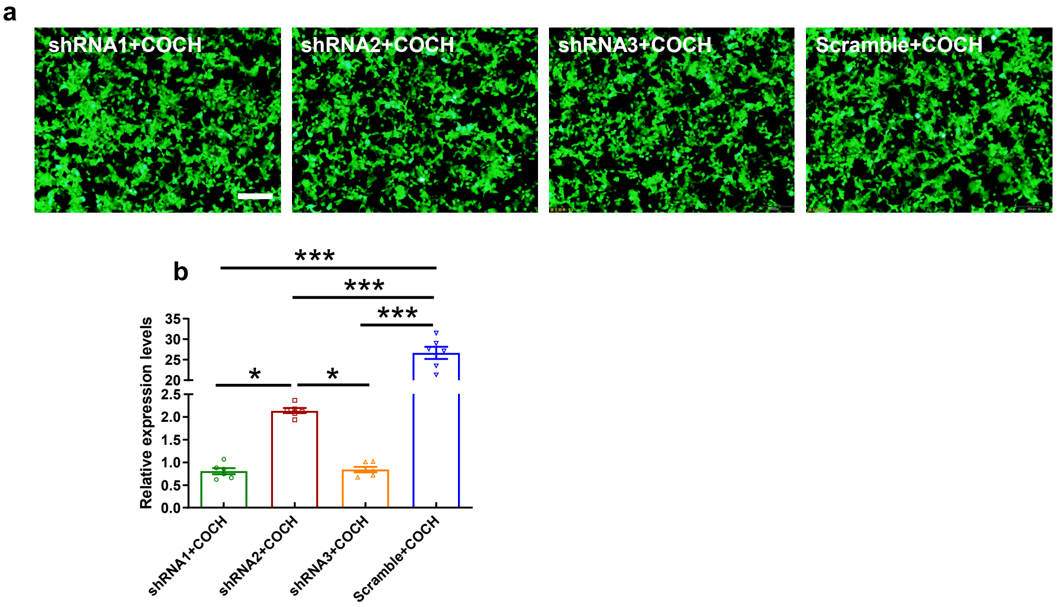

Supplementary Fig. 7 Selection of the optimal shRNA. Representative pictures of green fluorescent protein following cotransduction of 293T cells with lentiviruses carrying the guinea pig *Coch* gene and the shRNAs against its transcript (a). These pictures indicated a similar transduction efficiency of the cotransduced lentivirus overexpressing the guinea pig *Coch* gene and that expressing individual shRNA. The relative expression levels of the *Coch* gene were measured by qPCR and compared by one-way ANOVA (n=6), and the results showed that shRNA1 was the most efficient in knocking down *Coch* gene expression (b) and thus was selected for the following experiments in the FDM model. Scale bar = 200 μm. * $P < 0.05$, *** $P < 0.001$.

S. 8

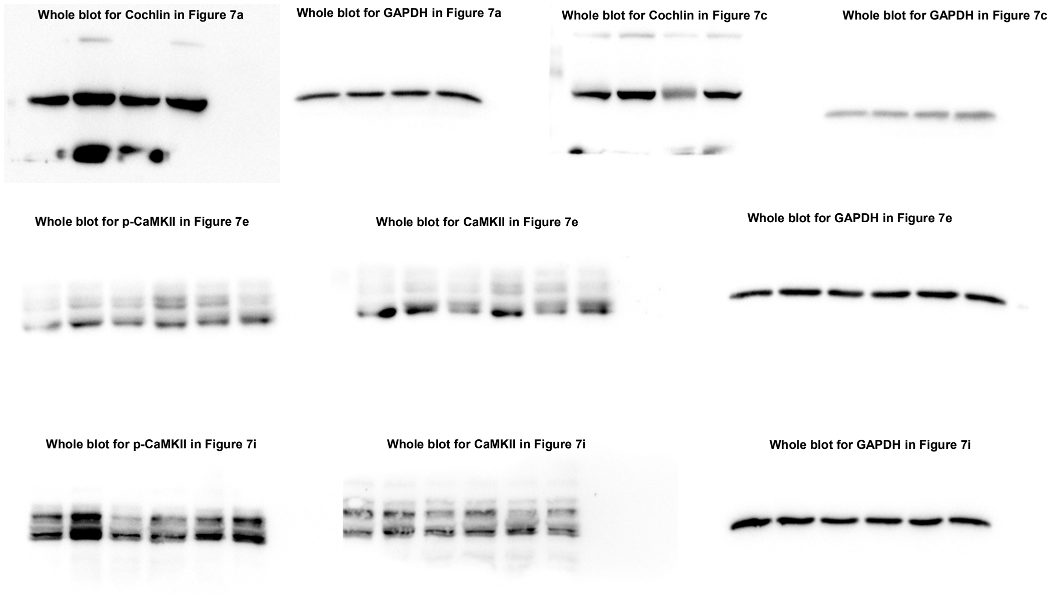

Supplementary Fig. 8 Whole blots for Figure 7a, c, e, and i.

Supplementary tables

Supplementary Table 1. Top 10 differentially expressed proteins between the FDM and NOR groups

Gene symbol	Gene description	Protein symbol	Fold change (FDM/NOR)	P Value
Coch	cochlin	COCH	3.78	0.001
Fhl1	four and a half LIM domains 1	FHL1	3.10	0.009
Krt19	keratin, type I cytoskeletal 19	KRT19	2.81	0.001
Krt4	keratin 4	KRT4	2.78	0.011
Krt8	keratin 8	KRT8	2.54	0.002
Map1b	microtubule associated protein 1B	MAP1B	0.47	0.001
Gfap	glial fibrillary acidic protein	GFAP	0.52	0.007
Sorbs2	sorbin and SH3 domain containing 2	SORBS2	0.52	0.008
Thbs1	thrombospondin 1	THBS1	0.57	0.025
Ncam1	neural cell adhesion molecule 1	NCAM1	0.61	0.017

Supplementary Table 2. Top 10 differentially expressed genes between the Cochlin and NOR groups

Phalanx ID	Gene symbols	Log_2 (Cochlin/NOR)	Annotations
PH_hs_0000753	*SFRP1*	8.61	Homo sapiens secreted frizzled-related protein 1
PH_hs_0063588	*GRB10*	7.73	Homo sapiens growth factor receptor-bound protein 10
PH_hs_0042289	*ZNF131*	7.68	Homo sapiens zinc finger protein 131
PH_hs_0035295	*RGPD6*/*RGPD5*	7.23	Homo sapiens RANBP2-like and GRIP domain containing 6/Homo sapiens RANBP2-like and GRIP domain containing 5
PH_hs_0044143	*TTLL9*	3.36	Homo sapiens tubulin tyrosine ligase-like family member 9
PH_hs_0042942	*RBM12B-AS1*	-10.38	Homo sapiens RBM12B antisense RNA 1
PH_hs_0006855	*METTL4*	-7.32	Homo sapiens methyltransferase like 4
PH_hs_0040813	*GRIK5*	-5.17	Homo sapiens glutamate receptor, ionotropic, kainate 5
PH_hs_0033892	*C2orf78*	-5.11	Homo sapiens chromosome 2 open reading frame 78
PH_hs_0004707	*FAM134B*	-4.91	Homo sapiens family with sequence similarity 134, member B

Supplementary Table 3. The antibodies used in this study

Antibodies	Species	Characteristics	Applications	Dilutions	Company	Catalog
Anti-cochlin	Rabbit	Primary/pAb	WB/IHC/IF	1:250/2000/ 1000	Abcam	ab171410
Anti-Rhodopsin	Mouse	Primary/mAb	IF	1:1000	Abcam	ab5417
Anti-p-CaMKII (Thr286)	Rabbit	Primary/mAb	WB	1:1000	CST	12716
Anti-GAPDH	Rabbit	Primary/mAb	WB	1:1000	CST	2118
Anti-CaMKII	Rabbit	Primary/pAb	WB	1:1000	CST	3362
Anti-p-β-catenin (Ser33/37/Thr41)	Rabbit	Primary/pAb	WB	1:1000	CST	9561
Anti-β-catenin	Rabbit	Primary/mAb	WB	1:1000	CST	8480
HRP-Anti-rabbit	Goat	Secondary	WB/IHC	1:2000	Abcam	ab6721
HRP-Anti-mouse	Goat	Secondary	WB	1:5000	Abcam	ab6789
Alexa-647-Anti-mouse	Goat	Secondary	IF	1:2000	Abcam	Ab150115
Alexa-488-anti-rabbit	Goat	Secondary	IF	1:2000	Abcam	Ab150077

Note: mAb: monoclonal antibody; pAb: polyclonal antibody; CST: Cell Signaling Technology; WB: western blot; IHC: immunohistochemistry; IF: immunofluorescence.

Supplementary Table 4. The oligonucleotides used in this study

Genes	Species		Sequences
Coch	Guinea pig	UP	5’-GATGGTCTGCTTCTTTCACAGTAACT-3’
		LP	5’-ACGCGCCGTGGACACT-3’
Actb	Guinea pig	UP	5’-ACGGAGCGTGGCTACAGTT-3’
		LP	5’-TCCTTGATGTCACGCACAATTT-3’
SFRP1	Human	UP	5’-CTGCCACCAGCTGGACAAC-3’
		LP	5’-CAGCAAGTACTGGCTCTTCACCTT-3’
GAPDH	Human	UP	5’-CCAGGTGGTCTCCTCTGACTTC-3’
		LP	5’-GTGGTCGTTGAGGGCAATG-3’
Coch	Guinea pig	shRNA1	5’-CTAATTGATGGAAGCTTTAATCTCGAGATTAAAGCTTCCATCAATTAG-3’
Coch	Guinea pig	shRNA2	5’-CAAAGTGGTGGTGGTATTTACTCGAGTAAATACCACCACCACTTTGG-3’
Coch	Guinea pig	shRNA3	5’-TGAGGGATAGTCCCAATAAACTCGAGTTTATTGGGACTATCCCTCAC-3’
Coch	Guinea pig	scramble	5’-CCTAAGGTTAAGTCGCCCTCGCTCGAGCGAGGGCGACTTAACCTTAGG-3’

Note: UP: upper primer; LP: lower primer.
