# Supplementary material for: Targeting the cochlin/SFRP1/CaMKII axis in the ocular posterior pole prevents the progression of nonpathologic myopia
Source: Commun Biol. 2023 Aug 29;6:884. doi: 10.1038/s42003-023-05267-2 (PMC10465513; doi:10.1038/s42003-023-05267-2)
Supplement: Supplementary file 3 — Description of Additional Supplementary Files [file 42003_2023_5267_MOESM3_ESM.pdf]

## Description of Additional Supplementary Files

**File name:** Supplementary Data 1
**Description:** The source data underlining the main figures presented in the manuscript.
