# Supplementary material for: Targeting the cochlin/SFRP1/CaMKII axis in the ocular posterior pole prevents the progression of nonpathologic myopia
Source: Commun Biol. 2023 Aug 29;6:884. doi: 10.1038/s42003-023-05267-2 (PMC10465513; doi:10.1038/s42003-023-05267-2)
Supplement: Supplementary file 5 — Reporting Summary [file 42003_2023_5267_MOESM5_ESM.pdf]

nature portfolio

Corresponding author(s): DBPR COMMSBIO-22-3491C

Last updated by author(s): Aug 8, 2023

Reporting Summary

Nature Portfolio wishes to improve the reproducibility of the work that we publish. This form provides structure for consistency and transparency in reporting. For further information on Nature Portfolio policies, see our Editorial Policies and the Editorial Policy Checklist.

Statistics

For all statistical analyses, confirm that the following items are present in the figure legend, table legend, main text, or Methods section.

n/a	Confirmed	
☐	☒	The exact sample size (*n*) for each experimental group/condition, given as a discrete number and unit of measurement
☐	☒	A statement on whether measurements were taken from distinct samples or whether the same sample was measured repeatedly
☐	☒	The statistical test(s) used AND whether they are one- or two-sided<br>*Only common tests should be described solely by name; describe more complex techniques in the Methods section.*
☒	☐	A description of all covariates tested
☐	☒	A description of any assumptions or corrections, such as tests of normality and adjustment for multiple comparisons
☒	☐	A full description of the statistical parameters including central tendency (e.g. means) or other basic estimates (e.g. regression coefficient) AND variation (e.g. standard deviation) or associated estimates of uncertainty (e.g. confidence intervals)
☐	☒	For null hypothesis testing, the test statistic (e.g. *F*, *t*, *r*) with confidence intervals, effect sizes, degrees of freedom and *P* value noted<br>*Give P values as exact values whenever suitable.*
☒	☐	For Bayesian analysis, information on the choice of priors and Markov chain Monte Carlo settings
☒	☐	For hierarchical and complex designs, identification of the appropriate level for tests and full reporting of outcomes
☒	☐	Estimates of effect sizes (e.g. Cohen's *d*, Pearson's *r*), indicating how they were calculated

Our web collection on statistics for biologists contains articles on many of the points above.

Software and code

Policy information about availability of computer code

Data collection	Excel 2019, CellSens Standard electronic system, Nanodrop 2000, RTVue XR Avanti,
Data analysis	SPSS 20.0, Graphpad Prism 8.0, ImageJ, ProteinPilot 4.0, R 3.0.3, amap, ctc, gplots, FlowJo, Python 3.7.9,

For manuscripts utilizing custom algorithms or software that are central to the research but not yet described in published literature, software must be made available to editors and reviewers. We strongly encourage code deposition in a community repository (e.g. GitHub). See the Nature Portfolio guidelines for submitting code & software for further information.

Data

Policy information about availability of data

All manuscripts must include a data availability statement. This statement should provide the following information, where applicable:

- Accession codes, unique identifiers, or web links for publicly available datasets
- A description of any restrictions on data availability
- For clinical datasets or third party data, please ensure that the statement adheres to our policy

The original proteomic data were uploaded to the website of iPoX and were assigned an ID of IPX0005328000, the data can be viewed through the link https://www.iprox.cn/page/PSV023.html;?url=1681372905604DkA1, with the password "iHSu". The original microarray data were uploaded to the website of ArrayExpress with an accession ID of E-MTAB-12364, these data can be viewed through the link https://www.ebi.ac.uk/biostudies/arrayexpress/studies/E-MTAB-12364?key=07f125bd-089e-4809-800e-ac7ae892de92.

Human research participants

Policy information about studies involving human research participants and Sex and Gender in Research.

Reporting on sex and gender	*Use the terms sex (biological attribute) and gender (shaped by social and cultural circumstances) carefully in order to avoid confusing both terms. Indicate if findings apply to only one sex or gender; describe whether sex and gender were considered in study design whether sex and/or gender was determined based on self-reporting or assigned and methods used. Provide in the source data disaggregated sex and gender data where this information has been collected, and consent has been obtained for sharing of individual-level data; provide overall numbers in this Reporting Summary. Please state if this information has not been collected. Report sex- and gender-based analyses where performed, justify reasons for lack of sex- and gender-based analysis.*
Population characteristics	*Describe the covariate-relevant population characteristics of the human research participants (e.g. age, genotypic information, past and current diagnosis and treatment categories). If you filled out the behavioural & social sciences study design questions and have nothing to add here, write "See above."*
Recruitment	*Describe how participants were recruited. Outline any potential self-selection bias or other biases that may be present and how these are likely to impact results.*
Ethics oversight	*Identify the organization(s) that approved the study protocol.*

Note that full information on the approval of the study protocol must also be provided in the manuscript.

Field-specific reporting

Please select the one below that is the best fit for your research. If you are not sure, read the appropriate sections before making your selection.

☒ Life sciences ☐ Behavioural & social sciences ☐ Ecological, evolutionary & environmental sciences

For a reference copy of the document with all sections, see nature.com/documents/nr-reporting-summary-flat.pdf

Life sciences study design

All studies must disclose on these points even when the disclosure is negative.

Sample size	According to ARRIVE guidelines, a power analysis shows that the sample size of 7-8 guinea pigs/group has a 90% power to detect a 230% change in the Refraction over a 6-week myopia induction period, assuming a 5% significance level and a two-sided test. However, the tri-color guinea pigs at 3 weeks of age have a relative high incidence of eye diseases, such as myopia and anisometropia greater than 1.5 D, therefore after excluding the guinea pigs with the eye diseases, only 40-50% of the purchased animals can be included in the experiments, we had to purchased 20 animals/group, in order to get the expected power of statistical significance.
Data exclusions	No data were excluded.
Replication	The animal experiments were independently repeated 3-4 times. The cell culture experiments were independently repeated 3 times.
Randomization	Each included animal was numbered, and the animal was allocated into an experimental group using random number table.
Blinding	Following the random grouping, the experimental groups were designated as "A, B, C, and etc.". Only the experiment designers knew which group the designation stood for. Neither the experiment performers nor the data analyzers were aware of the experiment grouping.

Reporting for specific materials, systems and methods

We require information from authors about some types of materials, experimental systems and methods used in many studies. Here, indicate whether each material, system or method listed is relevant to your study. If you are not sure if a list item applies to your research, read the appropriate section before selecting a response.

Materials & experimental systems

n/a	Involved in the study
☐	☒ Antibodies
☐	☒ Eukaryotic cell lines
☒	☐ Palaeontology and archaeology
☐	☒ Animals and other organisms
☒	☐ Clinical data
☒	☐ Dual use research of concern

Methods

n/a	Involved in the study
☒	☐ ChIP-seq
☐	☒ Flow cytometry
☒	☐ MRI-based neuroimaging

Antibodies

Antibodies used	All the antibody information is provided in Table 3 with the main manuscript file.
Validation	Primary Ab to Cochlin, the synthetic peptide used as the immunogen is within human Cochlin aa 398-428 region, which is identical between human and guinea pig Cochlin. This antibody has been validated to react specifically with human Cochlin. The other primary antibodies have all been validated by the manufacturers to specifically react to rodent target proteins.

Eukaryotic cell lines

Policy information about cell lines and Sex and Gender in Research

Cell line source(s)	RF/6A, an endothelial cell line derived from choroid and retina vessels of rhesus monkey.
Authentication	Short Tandem Repeat profiling has been performed to authenticate the cell line.
Mycoplasma contamination	The cell line has been tested negative for mycoplasma contamination.
Commonly misidentified lines (See ICLAC register)	HUVEC. This cell line represents the endothelial cells of peripheral vascular system, and is not suitable for the study of the vessels in the eye.

Animals and other research organisms

Policy information about studies involving animals; ARRIVE guidelines recommended for reporting animal research, and Sex and Gender in Research

Laboratory animals	Male colored guinea pigs of 3 weeks of age, Cavia porcellus, English short-hair stock, tricolor strain.
Wild animals	The study did not involve wild animals.
Reporting on sex	The male guinea pigs were used based on the literature (PMID: 35783515; PMID: 34171364) using the same models for myopia study. Moreover, it has been reported from the human study that the myopic boys tend to have longer axial length than girls (PMID: 36045001), therefore, we chose the male guinea pigs to acquire more significant phenotypes of our animal models. Finally, the developing male guinea pigs are generally stronger than the female counterparts, hence reducing the accidental loss of our experimental animals during the 6-week experiment and treatment period. However, we do not think our conclusion regarding to the molecular mechanism is restricted to the males only, we could test our conclusion in the female guinea pigs if necessary.
Field-collected samples	The study did not involve the samples collected from the field.
Ethics oversight	All the experimental procedures conformed to the Guide for the Care and Use of Laboratory Animals published by the US National Institutes of Health and were approved by the Laboratory Animal Care and Use Committee of our university (permission number: SYXK2009-0001).

Note that full information on the approval of the study protocol must also be provided in the manuscript.

Flow Cytometry

Plots

Confirm that:

☒ The axis labels state the marker and fluorochrome used (e.g. CD4-FITC).

☒ The axis scales are clearly visible. Include numbers along axes only for bottom left plot of group (a 'group' is an analysis of identical markers).

☒ All plots are contour plots with outliers or pseudocolor plots.

☒ A numerical value for number of cells or percentage (with statistics) is provided.

Methodology

Sample preparation	RF/6A cells were seeded into 24-well plates (Corning Costar, Cambridge, MA, USA) at a density of 1 X 105 cells/well and were treated based on the corresponding grouping information (n = 8-12 /group). Twenty-four hours later, the cells were stained with FITC-conjugated annexin V (FITC Annexin V Apoptosis Kit, Thermo Fisher Scientific, Waltham, MA, USA) according to the manufacturer's protocol. The cells were fixed with 1% paraformaldehyde (Sigma-Aldrich, St. Louis, MO, USA),.
Instrument	The percentage of the cells positive for annexin V staining was analyzed by a FACSCalibur (BD Biosciences, San Jose, CA, USA).
Software	FlowJo software (Becton, Dickinson and Company, Ashland, OR, USA).
Cell population abundance	Twenty-thousand gated events were acquired for analysis. The RF/6A cells cultured under the complete culture media and not stained with annexin V served as a negative control and were used to set the frame termed as "Annexin V positive". The cell population inside the "Annexin V positive" frame was considered positive for FITC-annexin V staining.
Gating strategy	The gate was set according to the characteristic properties of the RF/6A cells in forward and side scatter. The forward and side scatters were set in a linear scale.

☒ Tick this box to confirm that a figure exemplifying the gating strategy is provided in the Supplementary Information.
